# Combined detection of CA15-3, CEA, and SF in serum and tissue of canine mammary gland tumor patients

**DOI:** 10.1038/s41598-021-85029-4

**Published:** 2021-03-23

**Authors:** Yuying Fan, Xiaoli Ren, Xuesong Liu, Dongmei Shi, Enshuang Xu, Shuang Wang, Yun Liu

**Affiliations:** 1 Department of Veterinary Clinic, College of Veterinary Medicine, Northeast Agricultural University, Harbin, 150000 Heilongjiang People’s Republic of China; 2 College of Veterinary Medicine, Henan University of Animal Husbandry and Economy, Zhengzhou, Henan 450046 People’s Republic of China; 3 Branch of Animal Husbandry and Veterinary, Heilongjiang Academy of Agricultural Sciences, Qiqihar, 161000 Heilongjiang People’s Republic of China

**Keywords:** Cancer, Breast cancer, Cancer screening

## Abstract

The purpose of this study is to evaluate the levels and clinical diagnosis value of CA15-3, CEA, and SF in canine mammary gland tumors (CMGTs). In this study, the levels of tissues/serum CA15-3, CEA, and SF in 178 CMGT patients or healthy dogs were determined by ELISA and qRT-PCR assay. CA15-3, CEA, and SF levels of the malignant tumor group were significantly higher than that of the benign tumor group and the healthy control group. In the malignant tumor group, CA15-3 held a sensitivity of 51.8%, a specificity of 93.9%, and an accuracy of 76.8%. The sensitivity, specificity, and accuracy of CEA were 44.6%, 84.1%, and 68.1% respectively. SF held a sensitivity of 62.5%, a specificity of 85.4%, and an accuracy of 76.1%. SF showed the highest sensitivity and CA15-3 showed the highest specificity. The sensitivity, specificity, and accuracy of the combined detection of the three biomarkers in malignant tumor groups were 80.4%, 78.0%, and 80.0%, respectively, therefore combined detection increased sensitivity and accuracy but decreased specificity. In conclusion, the combined detection of serum/tissue markers CA15-3, CEA, and SF may improve the detection sensitivity of CMGTs, providing reference value for clinical application.

## Introduction

Canine mammary gland tumor (CMGT) is the most common neoplasms and the second leading cause of tumor death among intact female dogs, next to skin tumors^1^. Approximately 50% CMGTs are considered malignant by histopathologic diagnosis which are aggression, metastasis, and recurrence^2^. Early diagnosis of the tumor is the most critical factor in the survival and treatment of mammary gland tumor patients. Therefore, it has become an urgent and tough clinical problem to look for tumor markers of high sensitivity and specificity for CMGTs.

Tumor markers (TM) are referred to as substances produced by tumor cells, which usually indicate the presence of a tumor and serve for the management of patients with cancer^3^. TM exists in the patient’s blood, tissue, body fluid and excretions which can be used to detect the existence and growth of a tumor^4^. Cancer Antigen 15-3 (CA15-3) is a form of mucin glycoproteins (MUC1) which is a transmembrane protein possessing variable numbers of tandem repeats of peptides modified by glycosylation and is higher expressed in breast cancer compared with normal tissue^5^. It has been reported that CA15-3 is the most widely used serum tumor markers in clinical screening and early diagnosis of human breast cancer^6^. Likewise, in veterinary medicine, it has also proven that clinically healthy dogs can be distinguished from those with mammary neoplasia by CA15-3 level^7^. Although the CA15-3 is considered to possess the advantage of high specificity to detect precursors of breast cancer, single CA15-3 detection has a high rate of missing in malignant breast tumors, especially in early tumor patients^8^. Therefore, to meet clinical needs, CA15-3 should be combined with other TM to assist in the diagnosis of breast cancer and metastases. Carcinoembryonic antigen (CEA) is a glycoprotein produced by normal cells of the gastrointestinal mucosa which is involved in intercellular adhesion^9^. CEA is one of the first identified tumor markers of human breast cancer which is associated with tumor progression^10^. In veterinary medicine, dogs with mammary gland tumors held higher serum levels of CEA compared to healthy dogs^11^. But as a non-specific tumor marker, CEA can also be used for the diagnosis and auxiliary diagnoses of colorectal cancer, lung cancer, and pancreatic cancer^9^. Because of the low sensitivity and specificity of CEA in malignant tumors such as breast cancer in the early diagnosis and other applications, CEA should be combined with other tumor markers in auxiliary diagnosis, evaluation of effectiveness, and prognosis of tumor^12^. Ferritin is a soluble protein, which provides an important role in intracellular storage of bio-available iron and a tiny amount of ferritin is found in the serum^13^. Serum ferritin (SF), which is the main index of iron storage, variably overexpressed in various tumors, is also one of the tumor markers^14^. Studies in animal models have illustrated that subcutaneously injected and excess dietary iron increase the possibility of mammary carcinogenesis and accelerated mammary gland tumor progression^15^. It is demonstrated that malignant tumors can synthesize and secrete SF, increasing the amount of SF^16,17^. It is reported that human breast cancer are characterized by a higher SF level, when compared with normal tissue^18^. However, these tumor markers for the detection of mammary gland tumor shows limited diagnostic sensitivity and specificity^14,19^. The detection of the combination of multiple specific tumor markers to improve the diagnosis of canine malignant mammary gland tumor specificity, sensitivity is of great importance in clinical diagnosis^20^. The present study aimed to investigate the diagnostic value of single detection and combined detection of serum CEA, CA15-3, and SF in CMGTs and their association with clinical characteristics, thus providing experimental basis for seeking a diagnostic method for CMGTs.

## Results

### Epidemiological characteristics of mammary gland tumor in dogs

**Figure. 1:**
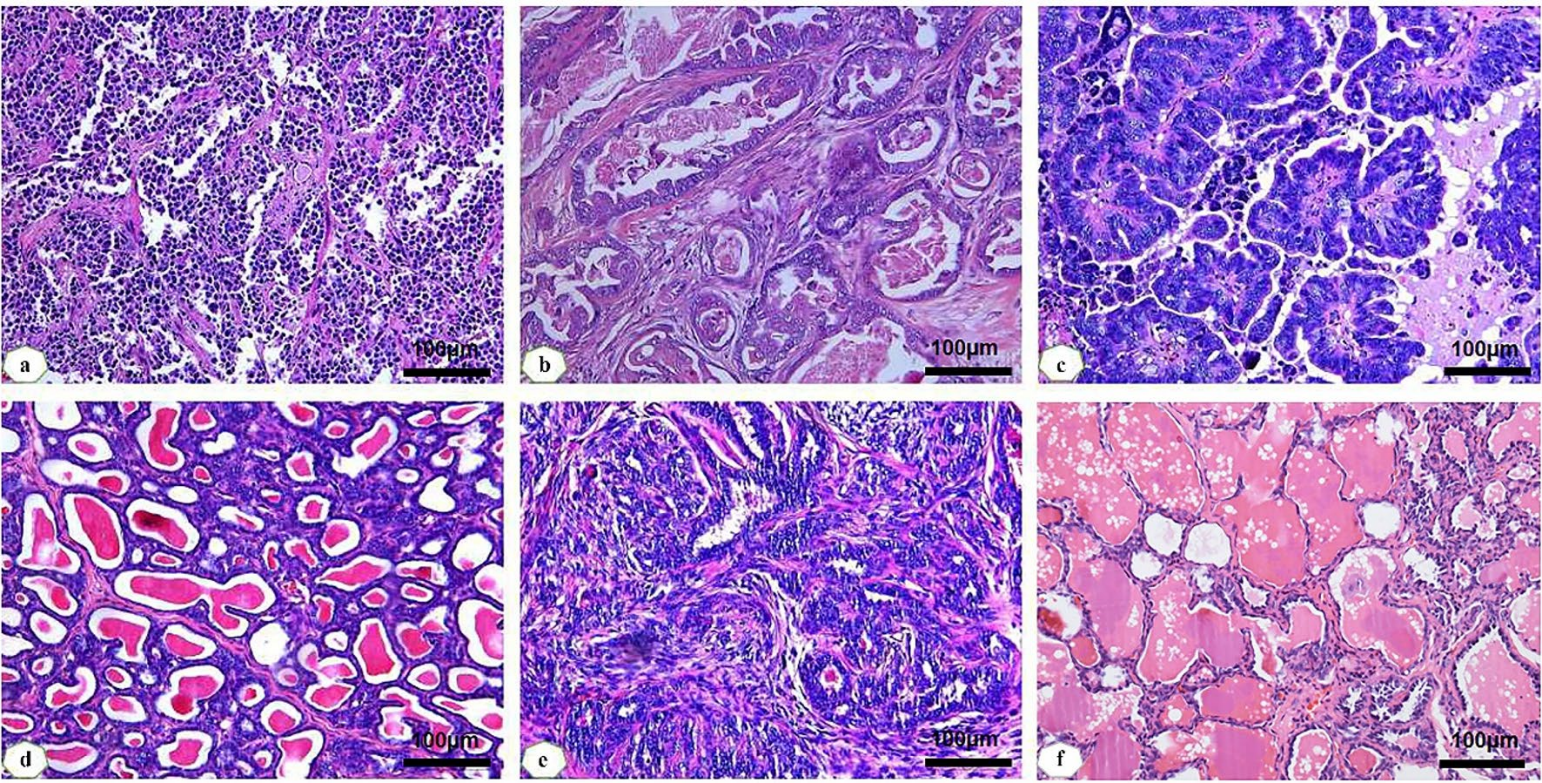
Histopathological observation of different mammary gland tumors in dogs (HE staining, ×100). **(a)** Invasive ductal carcinoma; **(b)** ductal carcinoma in situ; **(c)** micropapillary carcinoma; **(d)** adenopathy; **(e)** intraductal papilloma; **(f)** healthy mammary gland.

According to Table 1, among the 178 tissue samples, 40 were from healthy female dogs used as a control group with an age distribution between 3 and 15 years: average age 7.5 ± 2.4. There were 56 cases of malignant tumors, age ranging between 2 and 16 years: average age 10.6 ± 2.5. Benign tumor cases were 82, age ranging between 1 and 17 years, with an average age of 9.2 ± 3.4. The results of this study showed that the older the age, the higher the probability of mammary gland cancer, and the higher the degree of malignancy. Invasive ductal carcinoma (Fig. 1a), followed by micropapillary carcinoma (Fig. 1c), accounted for 53.6% and 12.5% of malignant tumors respectively. Typical histopathological morphology of ductal carcinoma in situ was showed (Fig. 1b). The most common types of benign tumors were intraductal papilloma (Fig. 1e) and adenosis (Fig. 1d), accounting for 31.7% and 23.2% of benign tumors respectively. The normal histological structure of canine mammary gland was showed (Fig. 1f).

**Table 1:** General clinical characteristic of mammary gland tumors with female dogs.

Clinical characteristics	Number	Frequency (%)
Control group	40	
**Ages**		
≥ 10	24	60
< 10	16	40
Benign tumor group	82	
**Ages**		
≥ 10	46	56.1
< 10	28	43.9
**Tumor size (cm)**		
T1 (< 3)	32	39
T2 (3 ≤ T ≤ 5)	27	32.9
T3 (> 5)	23	28.1
**Histological type**		
Intraductal papilloma	26	31.7
Adenopathy	19	23.2
Fibrosadenoma	15	18.3
Ductal and lobular hyperplasia	8	9.7
Other tumors	14	17.1
Malignant tumor group	56	
**Ages**		
≥ 10	23	41.2
< 10	33	58.8
**Tumor size (cm)**		
T1 (< 3)	12	21.4
T2 (3 ≤ T ≤ 5)	28	50
T3 (> 5)	16	28.6
**Histological type**		
Infiltrating ductal carcinoma	30	53.6
Ductal carcinoma in situ	5	8.9
Invasive micropapillary carcinoma	7	12.5
Intracystic papillary carcinoma	3	5.4
Intraductal papillary carcinoma	6	10.7
Others carcinoma	5	8.9
**Histologic grade**		
I	21	37.5
II	25	44.6
III	10	17.9

### Correlation between the expression of CA15-3, CEA, SF and clinicopathological parameters in dog serum with mammary gland cancer

**Figure. 2:**
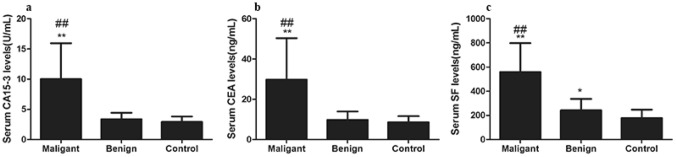
Expression levels of CA15-3, CEA, and SF in serum of canine mammary tumor. **(a)** Serum CA15-3 levels of malignant tumor group, benign tumor group, and healthy control group. **(b)** Serum CEA levels of the three groups. **(c)** Serum SF levels of the three groups. Note: * *P* < 0.05 showed significance difference, ** *P* < 0.01 showed extremely significant difference vs control group; # *P* < 0.05 showed significance difference, ## *P* < 0.01 showed extremely significant difference vs benign mammary gland tumor group.

Results showed that the serum levels of CA15-3, CEA, and SF in the malignant group was significantly higher than the healthy controls (*P* < 0.05). Although the levels of serum CA15-3 and SF in benign tumor groups were significantly higher than that of healthy controls (*P* < 0.05), both were in the normal range (Table 2, Fig. 2a,b). Serum CEA levels were no statistically significant between the benign tumor group and the healthy control group (*P* > 0.05). According to Table 2 and Fig. 2c, SF in malignant mammary gland tumor group was extremely significantly higher than that of benign mammary gland tumor group and healthy control group (*P* < 0. 01). The SF expression in the mammary gland benign tumor group was significantly higher than that of the healthy control group (*P* < 0.05). Univariate analysis showed that serum CA15-3, CEA, and SF concentration were extremely significantly different in dogs with lymph node invasion, metastasis, and histologic grading (*P* < 0.001; Table 2).

**Table 2:** Correlation between serum CA15-3, CEA and SF in canine mammary gland tumor and clinicopathological parameters.

Clinicopathological parameters	Number (%)	CA15-3 (U/mL)	CEA (ng/mL)	SF (ng/mL)
Age^*^				
≤ 10 years	33 (58.8%)	9.757 ± 5.832	28.52 ± 19.07	609.9 ± 236.5
> 10 years	23 (41.2%)	10.06 ± 5.850	26.20 ± 18.51	497.4 ± 217.3
*P*-value		0.335	0.203	0.863
Tumor location^*^				
Multiple	24 (42.4%)	10.11 ± 6.079	25.67 ± 18.08	549.5 ± 247.8
Single	32 (57.6%)	8.711 ± 4.651	28.99 ± 19.33	574.3 ± 224.9
*P*-value		0.067	0.445	0.201
**Tumor mass size**^#^				
T1 (< 3 cm)	12 (21.4%)	11.23 ± 6.506	28.29 ± 18.53	611.2 ± 224.8
T2 (3–5 cm)	28 (50.0%)	10.18 ± 5.779	26.21 ± 17.82	580.5 ± 244.8
T3 (> 5 cm)	16 (28.6%)	8.359 ± 5.044	29.39 ± 20.66	498.7 ± 211.4
*P*-value		> 0.05	> 0.05	> 0.05
Lymph node involvement^*^				
N0	41 (73.2%)	8.235 ± 5.364	21.88 ± 16.41	498.7 ± 227.3
N1	15 (26.8%)	14.39 ± 4.588	43.09 ± 16.27	738.8 ± 159.8
*P*-value		< 0.01	< 0.01	< 0.01
Metastasis^*^				
M0	44 (78.6%)	8.312 ± 4.747	22.83 ± 17.64	508.8 ± 222.8
M1	12 (21.4%)	15.64 ± 4.902	44.94 ± 11.76	765.1 ± 157.7
*P*-value		< 0.01	< 0.01	< 0.01
**Histologic grade**^#^				
I	21 (37.5%)	7.659 ± 4.705	20.72 ± 15.21	497.3 ± 199.9
II	25 (44.6%)	9.173 ± 5.114	27.09 ± 20.26	535.4 ± 238.3
III	10 (17.9%)	16.33 ± 5.103	43.14 ± 11.94	773.9 ± 169.9
*P*-value		< 0.05	< 0.05	< 0.05

^*^Unpaired t test; ^#^one ANOVA test; *P* < 0.05 show significant difference; *P* < 0.01 show extremely significant difference.

### Positive rate of serum tumor markers CA15-3, CEA, and SF

According to Table 3, the positive expression rates of serum tumor markers CA15-3, CEA, and SF were 51.8%, 44.6%, and 62.5%, respectively. The results showed that the positive rates of serum CA15-3, CEA, and SF were significantly different between the three groups (*P* < 0.05). The positive rate of serum CA15-3, CEA, and SF in the malignant group was significantly different from that in benign tumor group and healthy group (*P* < 0.05). However, there was no significant difference in the positive rates between the three groups of benign tumor group and healthy controls (*P* > 0.05).

**Table 3:** Positive expression rate of CA15-3, CEA and SF in serum.

Group	N	CA15-3 (U/mL)	CEA (ng/mL)	SF (ng/mL)
Malignant tumor group	56	29 (51.8%)	25 (44.6%)	35 (62.5%)
Benign tumor group	82	5 (6.1%)	13 (15.9%)	12 (14.6%)
Healthy control value	40	1 (2.5%)	1 (2.5%)	2 (5%)
χ^2^value		53.59	27.48	51.34
*P* value		< 0.01	< 0.01	< 0.01

*P* < 0.01 show extremely significant difference by χ^2^ test.

### Sensitivity, specificity, accuracy and Youden index of single and combined detections of serum CA15-3, CEA and SF in the diagnosis of Canine mammary gland malignant tumor

According to Table 4, among single detection of tumor biomarkers CA15-3, CEA, and SF, the specificity of CA15-3 was the highest, which is 93.9%, and followed by SF and CEA, which was 85.1 and 84.1 respectively. SF held the highest sensitivity, which was 62.5, and CA15-3 and CEA held lower sensitivities of 51.8 and 44.6 respectively. The Youden index represents the total ability of a screening method to discover real patients and non-patients. The larger the index, the better the effect of the screening experiment, the greater the authenticity. The Youden index of SF and CA15-3 were higher, which were 0.479 and 0.457, respectively, while CEA’s was lower, which was 0.287. Combined detection of biomarkers CA15-3, CEA, and SF held a sensitivity, accuracy, and Youden index 80.4%, 80.0%, and 0.584, respectively, which were all higher than that of any single detection. However, specificity in combined detection (78.0%) was lower than that of any single detection. These results suggested that the diagnostic rate of combined detection was higher than that of single detection and the combined detection of these three biomarkers may be useful in the diagnosis of CMGTs.

**Table 4:** Sensitivity, specificity, accuracy, and Youden index of single and combined detections of serum CA15-3, CEA and SF levels in canine mammary gland tumors.

Tumor markers	Sensitivity (%)	Specificity (%)	Accuracy (%)	Youden index (%)
CA15-3	51.8 (29/56)	93.9 (77/82)	76.8 (106/138)	0.457
CEA	44.6 (25/56)	84.1 (69/82)	68.1 (94/138)	0.287
SF	62.5 (35/56)	85.4 (70/82)	76.1 (105/138)	0.479
CA15-3 + CEA	64.2 (36/56)	81.7 (67/82)	74.6 (103/138)	0.459
CA15-3 + SF	71.4 (40/56)	84.1 (69/82)	78.9 (109/138)	0.555
CEA + SF	66.1 (37/56)	79.2 (65/82)	73.9 (102/138)	0.453
CA15-3 + CEA + SF	80.4 (45/56)	78.0 (64/82)	80.0 (109/138)	0.584

### Determination of area under the ROC of CA15-3, CEA and SF after single and combined detection

**Figure. 3:**
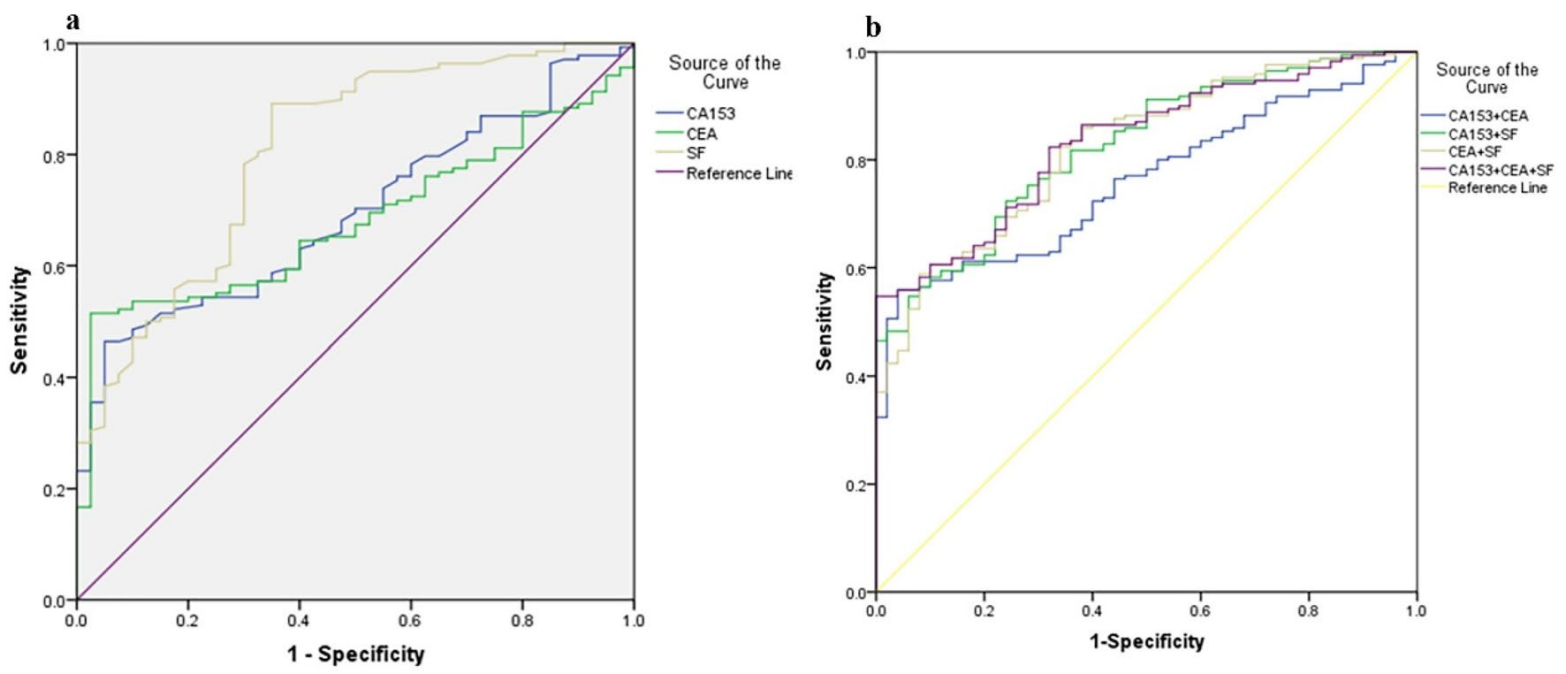
The ROC curve of single and combined detection in the diagnosis of malignant canine mammary gland tumor. **(a)** The ROC curves of single detection of CA15-3, CEA, and SF. **(b)** The ROC curves of combined detection of CA153 + CEA, CA135 + SF, CEA + SF, and CA153 + CAE + SF.

In order to assess the value of tumor markers in the diagnosis of CMGTs, it is necessary to use the ROC curve and to determine the area under the curve (AUC). According to Table 5 and Fig. 3a, each tumor marker has a significant value in the diagnosis of canine malignant mammary gland tumor (AUC > 0.5). SF (AUC = 0.810) showed the highest AUC in single detection, followed by CA15-3 (AUC = 0.751), the lowest was CEA (AUC = 0.680). According to Fig. 3b, among two tumor markers combined detection, CA15-3 + SF showed the highest AUC (AUC = 0.834) followed by SF + CEA (AUC = 0.814) the lowest was CEA + CA15-3 (AUC = 0.756). As for the combined detection of all three tumor markers, AUC was 0.838 higher than that of any two tumor markers combined detection. The combined detection of biomarkers showed a much higher diagnostic rate than single detection.

**Table 5:** The area under ROC curve of CEA, CA15-3 and SF in the diagnosis of Canine mammary gland tumor.

Serum tumor markers	AUC	P	95% CI
CA15-3	0.751	P < 0.001	0.679–0.823
CEA	0.680	P < 0.001	0.602–0.758
SF	0.810	P < 0.001	0.735–0.884
CA15-3 + CEA	0.756	P < 0.001	0.685–0.826
CA15-3 + SF	0.834	P < 0.001	0.774–0.898
CEA + SF	0.814	P < 0.001	0.742–0.886
CA15-3 + CEA + SF	0.838	P < 0.001	0.776–0.900

### Determination levels of CA15-3, CEA, and SF mRNA of tumor tissue samples

**Figure. 4:**
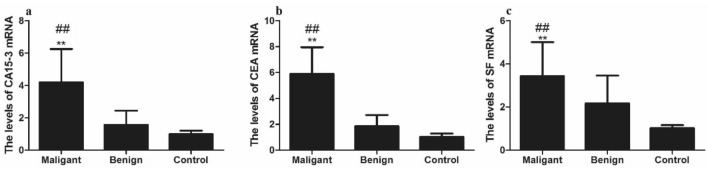
Levels of tissue CA15-3, CEA and SF mRNA in canine mammary gland tumor. **(a)** Tissue CA15-3 mRNA levels of malignant tumor group, benign tumor group, and healthy control group. **(b)** Tissue CEA mRNA levels of the three groups. c. Tissue SF mRNA levels of the three groups. Note: * *P* < 0.05 showed significance difference, ** *P* < 0.01 showed extremely significant difference vs control group; Comparison of benign mammary gland tumor group and healthy control group,# *P* < 0.05 showed significance difference,## *P* < 0.01 showed extremely significant difference vs benign mammary gland tumor group.

According to Fig. 4, the relative levels of CA15-3, CEA, and SF mRNA were higher in the malignant tumor group than that of benign tumor group and healthy control group with a significantly difference (*P* < 0.01). The relative levels of CA15-3 and CEA mRNA was not significant different between the benign tumor group and the healthy control group (*P* > 0.05). But the relative levels of SF mRNA were significantly different between the benign group and the healthy control group (*P* < 0.05).

## Discussion

In this study, malignant tumors accounted for approximately 40.1% of all CMGT cases, and the average age was 10.6 ± 2.5. In agreement with previous studies, there was a positive correlation between the age and the probability of malignant mammary gland tumor^21^. Surgical therapy is an effective treatment for CMGT, but its success or failure depends largely on the stage of the tumor. The earlier the stage, the higher the success rate. Therefore, early intervention and diagnosis can help reduce the fatality rate of CMGTs. To improve the detection rate of tumor and metastasis, TM is widely used for the early diagnosis of breast cancer in human medicine.

CA15-3 is recognized as the best tumor marker for the diagnosis of mammary gland cancer, which has a high value in tumor diagnosis and a positive correlation with the clinical stage^22^. Since the 1980s, it has been widely studied in human medicine, but there are still few reports on tumor biomarker research in veterinary medicine^23^. In this study, the expression of CA15-3 in malignant mammary tumor group was significantly higher than that in benign tumor group and healthy control group (*P* < 0. 01). CA15-3 held a sensitivity of 51.8% and a specificity of 93.9%. Previous studies found that the sensitivity and specificity of CA15-3 in breast cancer were 44.5% and 84.5% respectively and a significantly higher serum CA15-3 level had positive correlations with tumorigenesis and metastasis^24^. CEA is a glycoprotein and a broad-spectrum tumor marker which is mainly used in general the diagnosis of adenocarcinoma^9^. In this study, the expression of CEA in the malignant mammary gland tumor group was significantly higher than that of the benign group and healthy control group (*P* < 0. 01), however, there was no significant difference between the benign group and healthy control group (*P* > 0. 05). Similar results were found in studies investigating women with breast cancer, in which CEA levels were higher in women with breast cancer compared to healthy ones^25,26^. The sensitivity and specificity of CEA were 44.6% and 84.1% respectively, which were lower than that of a human mammary gland cancer research where sensitivity and specificity of CEA were 62.5% and 88.4% respectively^27^. Because of the low sensitivity and specificity of CEA, combined detection with other tumor markers is usually required^28^. Clinical studies have shown that SF is related to the occurrence and development of tumors because the iron utilization during tumorigenesis decreases, which leads to increased SF levels in tumor patients^29^. In the present study, the level of SF in the malignant mammary gland tumor group was significantly higher than that of the benign group and healthy control group (*P* < 0. 01). The sensitivity and specificity of SF were 62.5% and 85.4% respectively. Research on human medicine proved that SF level increased in patients diagnosed with malignant mammary gland tumor^29^. Also, hyperferritinemia was detected in canine with histiocytic sarcoma so that ferritin may be a useful serum tumor marker in dogs^16^. Nevertheless, it was showed that ferritin exhibited accumulation in benign lesions but not in malignant mammary gland tumors, while there was also evidence that ferritin was up-regulated in metastatic canine mammary tumors compared with orthotopic ones^30,31^. This may because different types of cancer cells hold distinct ability to produce ferritin, and further studies should be conducted on the mechanism of ferritin expression in cancer cells. The expression of CA15-3, CEA, and SF mRNA in canine malignant mammary gland tumor tissues was significantly higher than that in benign mammary gland tumor tissues and normal mammary gland tissues (*P* < 0.001). These results were similar to those obtained from blood serum. However, the sensitivity of single detection of CA15-3, CEA, and SF is low, which cannot match the need for clinical diagnosis.

This study also demonstrated that combined detection of CA15-3, CEA, and SF holds a higher sensitivity and accuracy than that of double and single detection. In the analysis of the ROC curve, the area under the ROC curve of SF single detection is greater than that of CA15-3 and CEA, indicating that SF is more effective than CA15-3 and CEA in the clinical diagnosis of CMGT. The area under the ROC curve of the combined detection of three tumor markers exceeded that of any single test, indicating that the diagnostic performance of the combined detection is better than that of a single test. A study on detection of breast cancer metastasis showed that combined detection of CA15-3 and CEA increased the sensitivity which proved that combined detection is more useful than single detection^32^. Again, it is reported that while CEA has a higher sensitivity of 75%, CA 15–3 has a higher specificity of 97%, the two tumor markers are complementary to each other, thus the combination of the two markers yielded a higher diagnostic accuracy^19^. However, it is worth noting that while the combined detection improves the sensitivity, the specificity decreases slightly. Therefore, when applying CA15-3, CEA and SF combined detection, it is necessary to pay attention to the false positive problem.

In conclusion, this study demonstrated that serum tumor markers CA15-3, CEA, and SF can be used as diagnostic tools for the screening of CMGTs. The sensitivity and accuracy of the combined detection of the three tumor markers were significantly higher than that of single detection. This is the first time that the combined detection of CA15-3, CEA, and SF is applied to the diagnosis of CMGTs. The results obtained suggest that the combined detection of tumor biomarkers CA15-3, CEA, and SF can be used as a method for the diagnosis of CMGTs and may increase the rate of diagnosis.

## Materials and method

### Ethical statement

Signed informed-consents were obtained from all the owners of dogs involved in this study. All the tissue samples and blood samples were obtained under professional veterinary care. Peripheral blood samples and tissue samples were collected from dog patients. No human participants were involved in the study. Approval for clinical trials was obtained from the Institutional Animal Care and Use Committee of Northeast Agricultural University, Harbin, China (approved by the State Council on October 31st, 1988 and promulgated by Decree No. 2 of the State Science and Technology Commission on November 14th, 1988).

### Collection of samples

Samples were collected from the veterinary hospital of the Northeast Agricultural University and other nearby hospitals around Harbin. A total of 178 samples of canine mammary gland tumors and normal tissue were identified during the period from 2014 to 2017. Among these, 56 cases were malignant between 2 and 16 years with an average age of 10.6 ± 2.5. 82 cases were classified as benign tumors, ranging from 1 to 17 years with an average age of 9.2 ± 3.4. 40 normal gland samples were derived from the normal adjacent tumor tissue of benign tumors. In addition, serum and tissue samples of 40 healthy dogs were used as a control group. Tumor patients were examined by the method of x-ray and B-ultrasound to exam tumor extent and any sign of distant metastases. Tissue samples were cut by a small part and then placed in appropriately labeled preservation jars containing liquid nitrogen. Most part of the samples fixed in 10% paraformaldehyde solution. After 24–72 h, samples were embedded in paraffin cut to 4–6 μm and stained in HE dye (Beijing Reagan Biotechnology Co., Ltd.). The tumors were classified based on the world health organization (WHO) TNM classification system according to Elston CW et al. and histological classification systems found by Goldschmidt et al.^33,34^. The histologic grade (I, II, III) of malignant tumor tissues were identified according to tubule formation, cell morphology, and mitotic rate. All tumor tissue samples were examined by two professional pathologists^33,34^. All animal procedures were approved and conducted in accordance with the Northeast Agriculture University IACUC.

### Determination of serum tumor biomarkers CA15-3, CEA, and SF levels

Peripheral blood samples (5 mL) were collected from clinically confirmed tumor patients and healthy dogs, stored at room temperature for 2 h, then centrifugated at 3500 *g* for 10 min. The serum was dispensed in 200 μL PCR tubes and stored at − 80∘. Double antibody sandwich ELISA method was used for detecting serum biomarkers CA15-3, CEA and SF levels (Jiangsu Jingmei China), and the antibody binds specifically to canine counterparts. The operation shall be carried out in accordance with the kit instructions. Epoch enzyme labeling instrument was produced by Bio Tek Company of the USA. The detection limits of the three markers were CA15-3 (0.1–5 U/mL), CEA (0.5–14 ng/mL) and SF (10–320 ng/mL).

### Real-time fluorescence quantitative PCR (RT-PCR)

The relative level of tumor markers in tissue samples was determined using real-time fluorescence quantitative PCR (RT-PCR). Total RNA from each sample was isolated and 1 μg of each sample was reverse-transcribed into cDNA using a PrimeScript RT reagent Kit with gDNA Eraser (Perfect Real Time) (Takara, Dalian, China). Real-time PCR was performed with the SYBR Premix Ex Taq (Perfect Real Time) (Takara, Dalian, China) and the reaction system was recommended by the manufacturer. Real-time fluorescence quantitative PCR instrument was purchased from Roche Company of the United States. Primers were designed according to the gene sequence of canine, as showed in Table 6. Real-time fluorescence quantitative PCR Parameters were as follows:

1. Pre-denaturation: 95 ℃ 30 s 1 cycle, 95 ℃ 5 s 1 cycle;
2. PCR reaction: 58 ℃ 20 s, 72 ℃ 20 s, 95 ℃ 10 s, 45 cycles;
3. Dissolution curve analysis: 65 ℃ 60 s, 97 ℃ 1 s, 1 cycle;
4. Cooling: 37 ℃ 30 s, 1 cycle.

**Table 6:** PCR primers used in the present study.

Gene name	Upstream primer (5′-3′)	Downstream primer (3′-5′)
GAPDH	GCTGCCAAATATGACGACATCA	GTAGCCCAGGATGCCTTTGAG
MUC1(CA15-3)	CTGCTGGTGCTGGTCTGTGTTCTG	GGCTGCTGGGTTCGGGTTCAT
CEACAM1(CEA)	GCCAGATTCTAACGCTCACGGATAG	AATCATCTTCCACATCCAGCCTTACAG
FER(SF)	GATGCTGCTTCTGGTATGTCCTATCTC	GAATACACTCCACCATCCTCTTGACG

GAPDH was acted as an internal parameter, and data was shown as 2^−△△Ct^ which calculated as △△Ct = (Ct Experimental group target gene − Ct Experimental group internal parameter gene) – (Ct Control group target gene − Ct Control group internal parameter gene).

### Statistical analysis

Statistical analysis was performed using SPSS 22.0 software and Graph pad prism (version 5.0, GraphPad Software Inc., San Diego, CA, USA). Using the t-test and χ^2^ test, data were expressed as mean ± standard deviation (x ± SD). Statistical differences were determined using one-way ANOVA. *P* < 0.05 was statistically significant. *P* < 0.01 showed an extremely significant difference. The sensitivity, specificity, accuracy, and Youden index of tumor markers in the diagnosis of Canine mammary gland tumors are as follows:

$${\text{Sensitivity }} = \, \left( {{\text{True positive }}/{\text{ Number of dogs diagnosed with malignant tumors}}} \right) \, \%$$

$${\text{Specificity}} = \, ({\text{True positive }}/{\text{ Number of dogs diagnosed with non-{malignant tumors}}}) \, \%$$

$${\text{Accuracy}} = \, ({\text{True positive}} + {\text{True negative}}) \, / \, ({\text{Number of dogs diagnosed with malignant tumor }} + {\text{ Number of dogs diagnosed with nonmalignant tumor}}) \, \%$$

$${\text{Youden index }} = \, ({\text{Sensitivity}}\,\,{\text{Specificity}}) \, - {1}$$

The boundary value of the tumor marker is defined by the method of operating characteristic curve of the subject or ROC curve. The higher the area under the curve (AUC) the higher the diagnostic value. Accuracy reaches the highest when AUC > 0.9. The value of specificity and sensitivity of tumor markers in canine mammary gland tumors is evaluated by using the ROC curve. The area under the curve (AUC) 1.0 is regarded as the most ideal index. There is no diagnostic value if AUC < 0.5.
